# Extreme Point Sort Transformation Combined With a Long Short-Term Memory Network Algorithm for the Raman-Based Identification of Therapeutic Monoclonal Antibodies

**DOI:** 10.3389/fchem.2022.887960

**Published:** 2022-04-13

**Authors:** Jin Ling, Luxia Zheng, Mingming Xu, Gang Chen, Xiao Wang, Danzhuo Mao, Hong Shao

**Affiliations:** ^1^ NMPA Key Laboratory for Quality Control of Therapeutic Monoclonal Antibodies, Shanghai Institute for Food and Drug Control, Shanghai, China; ^2^ NMPA Key Laboratory for Quality Analysis of Chemical Drug Preparations, Shanghai Institute for Food and Drug Control, Shanghai, China

**Keywords:** Raman spectroscopy, long-short term memory network, therapeutic monoclonal antibody, extreme point sort transformation, algorithm study

## Abstract

Therapeutic monoclonal antibodies (mAbs) are a new generation of protein-based medicines that are usually expensive and thus represent a target for counterfeiters. In the present study, a method based on Raman spectroscopy that combined extreme point sort transformation with a long short-term memory (LSTM) network algorithm was presented for the identification of therapeutic mAbs. A total of 15 therapeutic mAbs were used in this study. An in-house Raman spectrum dataset for model training was created with 1,350 spectra. The characteristic region of the Raman spectrum was reduced in dimension and then transformed through an extreme point sort transformation into a sequence array, which was fitted for the LSTM network. The characteristic array was extracted from the sequence array using a well-trained LSTM network and then compared with standard spectra for identification. To demonstrate whether the present algorithm was better, ThermoFisher OMNIC 8.3 software (Thermo Fisher Scientific Inc., U.S.) with two matching modes was selected for comparison. Finally, the present method was successfully applied to identify 30 samples, including 15 therapeutic mAbs and 15 other injections. The characteristic region was selected from 100 to 1800 cm^−1^ of the full spectrum. The optimized dimensional values were set from 35 to 53, and the threshold value range was from 0.97 to 0.99 for 15 therapeutic mAbs. The results of the robustness test indicated that the present method had good robustness against spectral peak drift, random noise and fluorescence interference from the measurement. The areas under the curve (AUC) values of the present method that were analysed on the full spectrum and analysed on the characteristic region by the OMNIC 8.3 software’s built-in method were 1.000, 0.678, and 0.613, respectively. The similarity scores for 15 therapeutic mAbs using OMNIC 8.3 software in all groups compared with that of the relative present algorithm group had extremely remarkable differences (*p* < 0.001). The results suggested that the extreme point sort transformation combined with the LSTM network algorithm enabled the characteristic extraction of the therapeutic mAb Raman spectrum. The present method is a proposed solution to rapidly identify therapeutic mAbs.

## Introduction

Biologics, especially therapeutic monoclonal antibodies (mAbs), have been progressively utilized in clinics over the last decade and have been established as a major therapeutic modality to treat severe human diseases (Singh et al., 2018; Li et al., 2020). To date, approximately 80 antibody-based biopharmaceutical products have been approved for therapeutic use worldwide in 2019, and 42 therapeutic mAbs have been marketed in China (Castelli et al., 2019). Over 100 mAbs are in development, and new therapeutic mAbs are marketed every year (Ettah and Ashton, 2018; Castelli et al., 2019). Meanwhile, the number of counterfeits is increasing. Therapeutic mAb products are commonly colourless injections or white freeze-dried powder, which are easy to make counterfeit (Loethen et al., 2015). Therapeutic mAbs are considered an attractive target for counterfeiters for another important reason: they are very expensive (Degardin et al., 2017). Therefore, strict control of therapeutic mAb products is required to fight against the counterfeit of mAbs (Frosch et al., 2019). Velpandian et al. did a protein analysis using Bradford assay and SDS-PAGE to confirm the presence of bevacizumab in 16 samples including six suspected and 10 others. The results suggested that the counterfeit bevacizumab can be preliminarily identified by simple methods (Velpandian et al., 2016). These simple methods can only differentiate the therapeutic mAbs from the protein-free counterfeits. Legrand et al. reported a strategy based on the concomitant use of capillary zone electrophoresis analysis (CZE-UV), size exclusion chromatography coupled to multi-angle light scattering (SEC-MALS) and liquid chromatography hyphenated to tandem mass spectrometry (LC-MS/MS) which enables the structural identification of mAbs in addition to comprehensive characterization and quantification in samples in potentially counterfeit samples (Legrand et al., 2022). Although the concomitant use of several analysis methods can provide an accurate result, it is complicated and takes a long time.

Various rapid analysis methods based on spectrum and chromatography can allow quick identification and quality assurance in pharmacies, medical facilities, pharmaceutical warehouses, etc., (Igne et al., 2015; Hattori et al., 2018; Tobias et al., 2021). These analysis methods usually take a few minutes to give a result, which will enhance public safety. In particular, Raman spectroscopy is fast and easy to operate and provides molecular fingerprints by detecting unique vibrations from atomic bonds within a molecular structure (McAvan et al., 2020). A Raman spectrum usually contains functional groups and an overall chemical arrangement that could be used for molecular structure analysis (Lanzarotta et al., 2020). As a result, the Raman technique has been widely employed in the pharmaceutical industry and in the random inspection of small molecule drugs (Lawson and Rodriguez, 2016). Several applications have been reported for mAb analysis. A Raman spectroscopy-based nutrient control strategy was developed to enable dual control of lactate and glucose levels for a fed-batch CHO cell culture process for mAb production, which could benefit both cell metabolism and mAb product quality (T et al., 2021). Yilmaz and his colleagues demonstrated the possibility of subclass-independent quantitative mAb prediction by Raman spectroscopy in real time (Yilmaz et al., 2020). The applicability of Raman spectroscopy to detect posttranslational modifications (PTMs) and degradation seen in mAbs in the manufacturing process has been investigated (McAvan et al., 2020). The reports highlighted Raman spectroscopy as a technique to suit the application of mAb analysis, as it is information-rich, minimally invasive, insensitive to water background and requires little to no sample preparation (Jones et al., 2019).

A long short-term memory (LSTM) network is a novel recurrent neural network (RNN) architecture in conjunction with an appropriate gradient-based learning algorithm (Yu et al., 2021). Unlike RNN networks, the LSTM network has a hidden inner state that enables applying the previous sequence input to a later calculation and avoids gradient disappearance through a gating unit system. Efficient processing of sequences of data and effective propagation of information along the sequence are commonly observed in LSTM networks. Therefore, LSTM networks have been successfully applied to process time-series and position-series data, for example, stemming from speech or video and natural language sentences. Some studies are currently attempting to use LSTM networks to analyse Raman spectra. Yu et al. presented Raman spectroscopy combined with an LSTM network to predict eight strains isolated from the marine organism *Urechis unicinctus* and compared them with a method using a normal convolutional neural network (CNN) (Yu et al., 2021). The proposed LSTM methods achieved average isolation-level accuracies greater than 94%, which is higher than that of the normal CNN network. Wang and his colleagues presented a rapid method to screen hepatitis B patients using serum Raman spectroscopy combined with LSTM networks. In a previous report, principal component analysis (PCA) was selected to extract key features of spectral data for dimensional reduction of the multidimensional spectrum, followed by training of the LSTM network. Meanwhile, a fully connected layer (FCL) was used for the classification of HBV (Wang et al., 2020).

Spectral characteristic extraction is very important in the signal preprocessing step (Fukuhara et al., 2019; Galeev et al., 2019; Ling et al., 2020). Original spectral data usually contain many invalid data points, which will cause a serious performance loss and low accuracy rate. Without preprocessing characteristic extraction, model training will be difficult. FCL is usually used as a classifier at the terminal of the model. However, artificial neural networks are nonlinear fitting models, and adding an FCL classifier may cause overfitting in some cases, especially in small-sample-size training. In the present study, we introduce a novel Raman spectrum detection approach with LSTM networks for therapeutic mAb identification. First, fast Fourier transformation (FFT) and inverse fast Fourier transformation (IFFT) are performed on the spectral characteristic region to remove spectral noise and reduce dimensions. Extreme point sort transformation is a critical step to convert the spectrum into a sequence array that involves the peak absolute positions and their relative intensities. The sequence array can be considered a number-labelled sentence, which fits the LSTM network. LSTM networks are well trained and then employed to extract the characteristics of the sequence array deeply into a characteristic array. The extracted characteristic array will be compared with the characteristic arrays extracted from standard spectra using the same method to obtain a similarity score. The sample will be identified as a therapeutic mAb class where the similarity score is higher than the threshold value of the therapeutic mAb class.

## Methods

### Therapeutic Monoclonal Antibodies

A total of 15 therapeutic mAbs from seven manufacturers, including adalimumab, bevacizumab, ranibizumab, tocilizumab, evolocumab, secukinumab, rituximab, trastuzumab, pertuzumab, denosumab, ixekizumab, ustekinumab, guselkumab, emicizumab, and etanercept, were selected for the study. Eleven effect targets and four protein formats of mAbs were involved when choosing therapeutic mAbs. Information on the 15 therapeutic mAbs is shown in Supplementary Table S1. They were labelled from 0 to 14 for artificial intelligence identification.

### Raman Analysis

**FIGURE 1:**
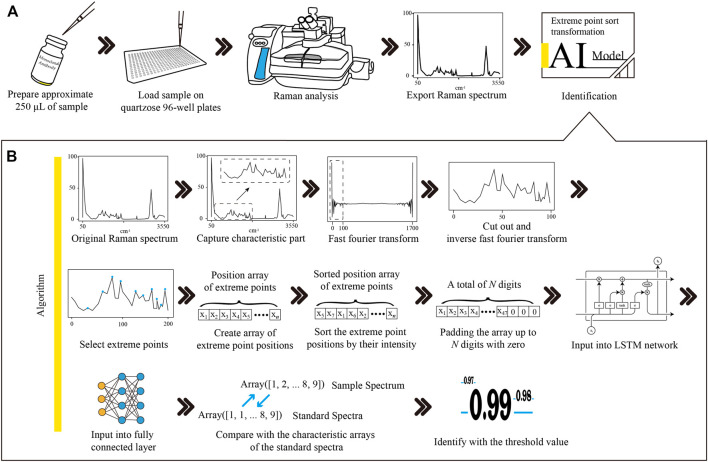
The workflow of Raman-based therapeutic mAb identification and the procedures of Raman spectrum transformation combined with LSTM network modelling. **(A)** The therapeutic mAb sample was loaded into a quartzose 96-well plate. Raman analysis was performed to obtain the Raman spectrum of the sample. The Raman spectrum was exported into the CSV file and then input into the LSTM network model for identification. **(B)** The characteristic region of the Raman spectrum was extracted from the original spectrum. The noises of the characteristic region were removed, and the dimension was reduced with FFT and IFFT filtering. The positions of the extreme points were picked up and then sorted by the intensity of each point to create an array. The array was padded up to *N* digits with zero to satisfy the requirement of the input layer of the LSTM network model. The LSTM layer sequentially connected to the FCL formed the hidden layers of the model. The similarity of the sample spectrum and the standard spectrum was calculated by comparing their outputs from the FCL. The sample was identified as the therapeutic mAb, and the similarity value was higher than the threshold. mAb, monoclonal antibody; LSTM, long short-term memory; CSV, comma separated value; FFT, fast Fourier transformation; IFFT, inverse fast Fourier transformation; FCL, fully connected layer.

As shown in Figure 1A, approximate 250 μL of therapeutic mAb injections were loaded into a 96-well quartzose plate for Raman scanning. Sufficient injection fluid had to be added to make the fluid level flush with the rim of the tiny cup. ThermoFisher Scientific DXR confocal Raman microscopy (Thermo Fisher Scientific Inc., U.S.) was employed for analysis, which was equipped with a 20× objective, a 532 nm laser and a 532 nm filter. A full spectral range of 57–3,417 cm^−1^ was set to acquire the Raman spectrum. Data acquisition was performed using ThermoFisher OMIC 8.3 software (Thermo Fisher Scientific Inc., U.S.) with a 10 mW laser power, 10 s exposure time, and 20 scans. For each therapeutic mAb, 18 samples in three batches were randomly selected to collect spectra. Raman spectra were acquired from five random points on each sample. Finally, a total of 1,350 Raman spectra were obtained to build a Raman spectrum database for 15 therapeutic mAbs.

### Dataset Preparation

Spectra were exported as comma-separated values (CSV) files using ThermoFisher OMIC 8.3 software. The CSV file contained the Raman shift values and the numeric value of the intensity for every single point of the Raman spectrum. These numeric values of both Raman shift and intensity in CSV files were read by Python v3.8.5 and then inserted into a MySQL v5.7.20 (MySQL AB, Sweden) database table to create an in-house dataset with some basic information, such as mAb name, specifications, and date of analysis. Numeric labels of data from 0 to 14 were assigned to each mAb. Before data processing, all numeric value data with labels were exported from the database into a text file to obtain high loading performance.

### Data Transformation

Here, we present an extreme point sort transform for the array of Raman spectra, which transforms array-type spectrum data into a list of sequence numbers. First, the characteristic part of the mAbs, ranging from 100–1,800 cm^−1^ in the Raman spectrum, was captured. Then, noise removal and dimensionality reduction were performed on the characteristic region using FFT and IFFT. The FFT formula is shown as follows:

$$X\left(k\right)=\overset{N-1}{\underset{n=0}{\sum }}x\left(n\right)e^{-j\left(\frac{2\pi }{N}\right)nk}\;\left(k=0,1,\ldots ,M-1\right)$$ (1)

where k is the consecutive integers from 0 to M −1, n is the sequence number of the time-domain signal array, and  M is the total number of array elements.

After FFT processing, the low-frequency range was picked to perform IFFT to obtain a noise removed and a low-dimensional spectrum. The IFFT formula is shown as follows:

$$\text{x}\left(\text{n}\right)=\frac{1}{N}\overset{N-1}{\underset{n=0}{\sum }}X\left(k\right)e^{j\left(\frac{2\pi }{N}\right)nk}\left(n=0,1,\;\ldots ,\;N-1\right)$$ (2)

where n is the consecutive integers from 0 to N −1, k is the sequence number of the frequency-domain signal array, and  N is the total number of the low-frequency range array elements.

The positions of extreme points of the spectrum were extracted using the Scipy library. The first extreme point was removed and then sorted by their intensities in descending order. The extreme point array was padded up to N digitals with zero to match the input dimension of the model.

Data visualization after each processing step was performed using the Matplotlib library. The data labels were converted into one-hot labels using the Keras library. The dataset containing all numeric values and labels was split randomly into a training dataset and validation dataset with a split ratio of 0.8, which means that 80% of the data were used for model training and the other 20% were used for model validation. The test dataset was created using 20 additional Raman spectra of each therapeutic mAb. These spectra were never used before as a test set.

### Long Short-Term Memory Modelling

**FIGURE 2:**
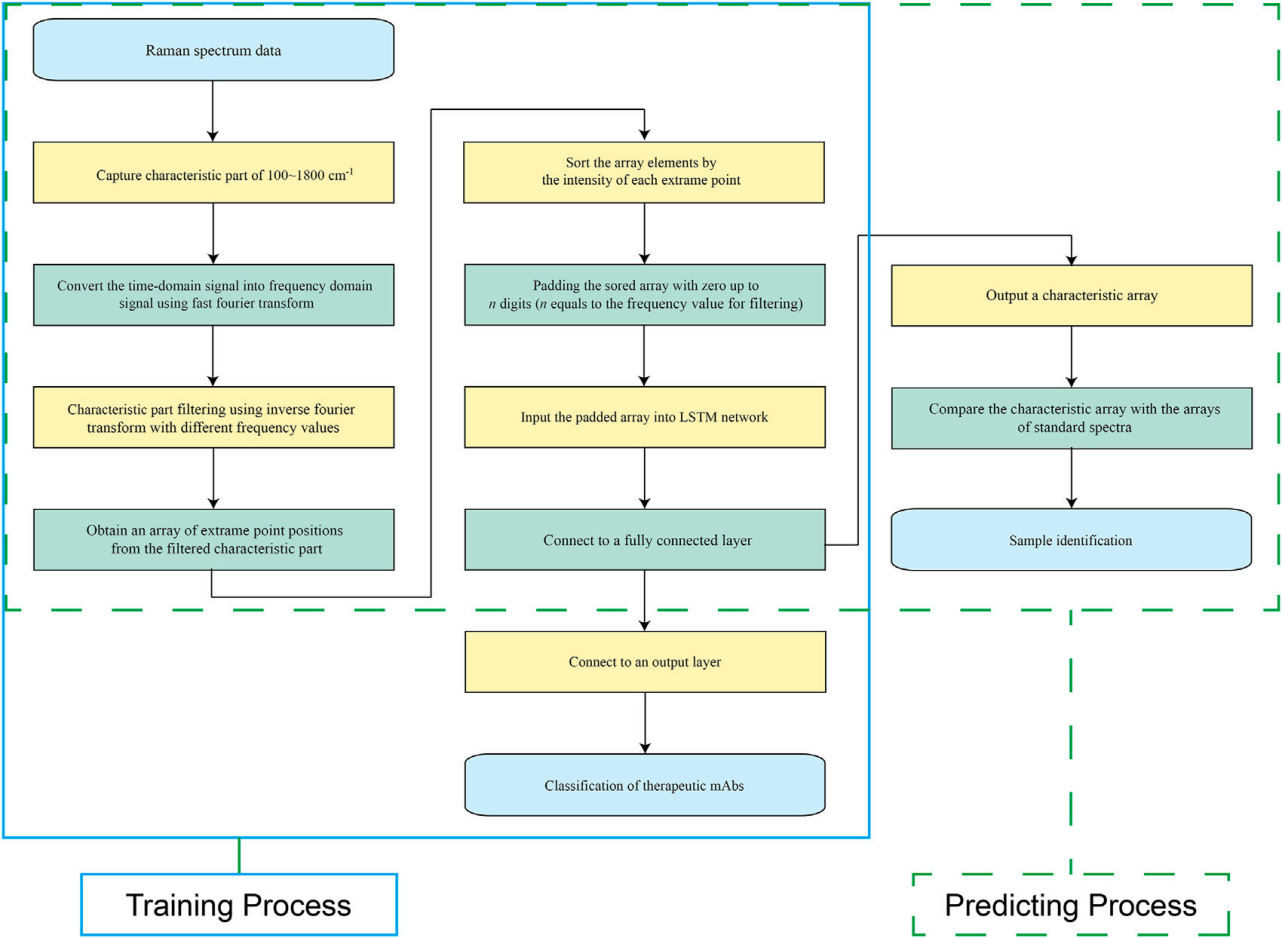
Schematic view of the training process and prediction process. The training process is marked with a light blue full line box, and the prediction process is marked with a green dashed line box.

As shown in Figure 2, the training process was marked within a light blue full line box, and the predicting process was marked within a green dashed line box. The well-trained models were employed for spectral characteristic extraction to obtain a characteristic array, which was compared to the characteristic arrays of standard spectra for final identification.

All training and predictions were carried out on a SuperMicro 7049GR GPU server equipped with Dual Intel(R) Xeon(R) Gold 6,149 CPUs, 128 Gb of DDR4 RAM and Quad Nvidia RTX 3090 24 Gb graphics cards. The operating system was the 64-bit CentOS Linux system v7.5. TensorFlow v2.0.0 was employed to train and validate the LSTM network models.

As shown in Supplementary Table S2, the LSTM network model contains one embedding layer, one bidirectional LSTM layer, one FCL, one dropout layer and one output layer to form the output prediction. The dimension of the LSTM layer is 64. The numbers of nodes in the FCL and output layer were 32 and 15, respectively. The activation function of the FCL and output layer were the rectified linear unit (ReLU) function and Softmax function, respectively. The softmax function defined in Eq. 3 was applied in the last layer to produce the prediction probability over the 15 output classes (Hsieh et al., 2020).

$$f{\left(s\right)}_{i}=\frac{e^{s_{i}}}{\sum_{j=1}^{C}e^{s_{j}}}$$ (3)

where $s_{i}$ are the scores inferred by the net for each class in C .

The categorical cross-entropy was selected as the loss function, which was defined in Eq. 4. The goal of the network is to minimize CE .

$$\text{CE}=-log\frac{e^{s_{p}}}{\sum_{j}^{C}e^{s_{j}}}$$ (4)

where $s_{p}$ is the score for the positive class.

Adam is selected as the optimizer. The learning rate was set as 0.001, and the number of epochs was set as one to five.

### Similarity Scoring

Characteristics of the Raman spectrum were extracted as a characteristic array using extreme point sort transformation and the LSTM network model. The characteristic array of the sample spectrum was compared with that of standard spectra to obtain the cosine similarity score using the Sklearn library. The threshold value of the cosine similarity score was set as the lowest score, which was calculated by comparing each standard spectrum to the remaining standard spectra. The sample spectrum was identified when the cosine similarity score was higher than the threshold value.

### Production of Simulative Spectra

Spectral peak drift, random noise and fluorescence interference were considered to test the robustness of the method. Elements in the spectral intensity array were integrally shifted to the left or right with 1–20 digitals to simulate spectral peak drift of 1–20 cm^−1^. The blank positions produced while shifting were filled with the adjacent value. For simulated random noise on the spectrum, every single element in the spectral intensity array was multiplied by a random percentage. The range of the random percentage is 60–100% or 100–140%. For simulated fluorescence interference, a positive half-sine part wave overlapped in the 0–1800 cm^−1^ range of the original spectrum. The sine wave amplitude was set as 60–140% of the maximum peak intensity. The signal frequency and sampling frequency were both set as 1. The sine wavelength was set as a random integer in the range of 3,600–7,200. The reverse arrangement of the elements in 0–1800 digital positions was picked up as a positive half-sine part to create the simulative spectrum.

### Evaluation of Prediction

The loss, precision, accuracy and recall were used to evaluate the model since they are commonly used in most cases for evaluations. The loss values were calculated using the categorical cross-entropy formula described above. The precision, accuracy and recall were calculated as follows:

$$Precision=\frac{tp}{tp+fp}$$ (5)

$$Accuracy=\frac{tp+tn}{tp+tn+fp+fn}$$ (6)

$$Recall=\frac{tp}{tp+fn}$$ (7)

where tp is true positives, fp is false-positives, tn is true negatives, and fn is false negatives.

A confusion matrix was established to investigate the classification performance. Each column of the matrix stands for a predicted label, while each row represents a true label. The receiver operating characteristic (ROC) curve was drawn with the true positive rate and false-positive rate.

Tests of the prediction performance for the present method and ThermoFisher OMNIC 8.3 software built-in method (analysed on full spectrum and characteristic region, respectively) were performed using a total of 300 Raman spectra (20 Raman spectra for each therapeutic mAb) test set with labels. The ThermoFisher OMNIC 8.3 software built-in method was used to match the spectrum with the user’s standard spectrum library. The match parameter of the analysis region was set as the full spectrum or a range of 100–1800 cm^−1^ for full spectrum analysis or characteristic region analysis, respectively.

### Sample Identification

A total of 30 samples, including 15 therapeutic mAb injections, three biochemical drug and biological product injections, and 12 chemical drug injections, were selected for the identification test using the present method. A sample is identified when the similarity score is higher than the threshold value of the standard spectrum. If the similarity score was lower than every threshold value, it was identified as “not matched”.

## Results

### Identification Approach

As shown in Figure 1, an optimized Raman identification approach for therapeutic mAbs was proposed. Approximately 250 μL of sample was loaded on a quartzose 96-well plate, followed by Raman analysis to obtain the spectrum. The spectral data were exported into a CSV file with 3,417 data points for data analysis.

### Characteristic Region Acquirement

**FIGURE 3:**
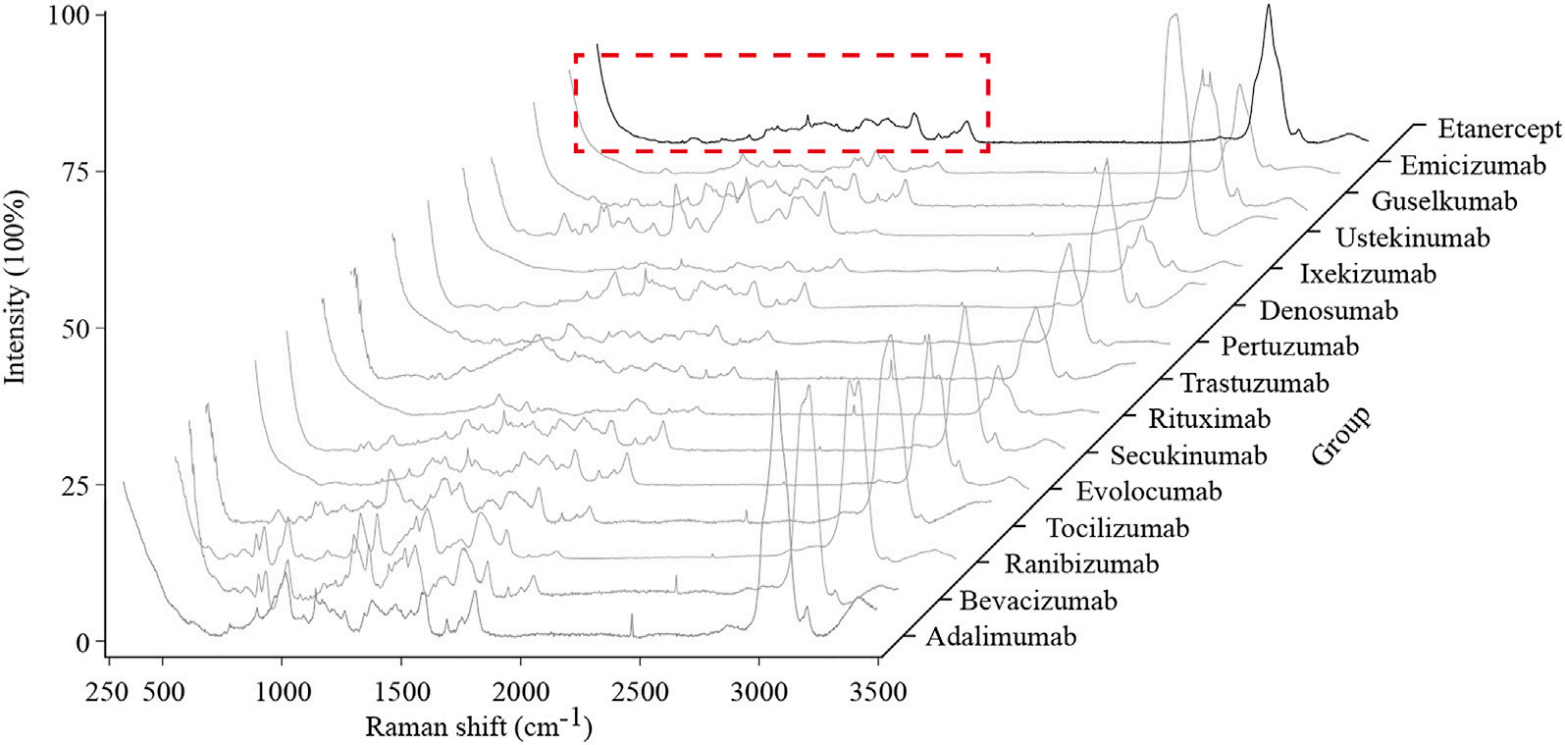
Typical Raman spectra of the 15 therapeutic mAbs. The characteristic region of the Raman spectrum is marked with a red dashed line box.

Fifteen typical spectra of therapeutic mAbs are shown in Figure 3. By comparing each spectrum manually, a range of 100–1800 cm^−1^ was selected as the characteristic region, which is information-rich.

### Data Transformation

**FIGURE 4:**
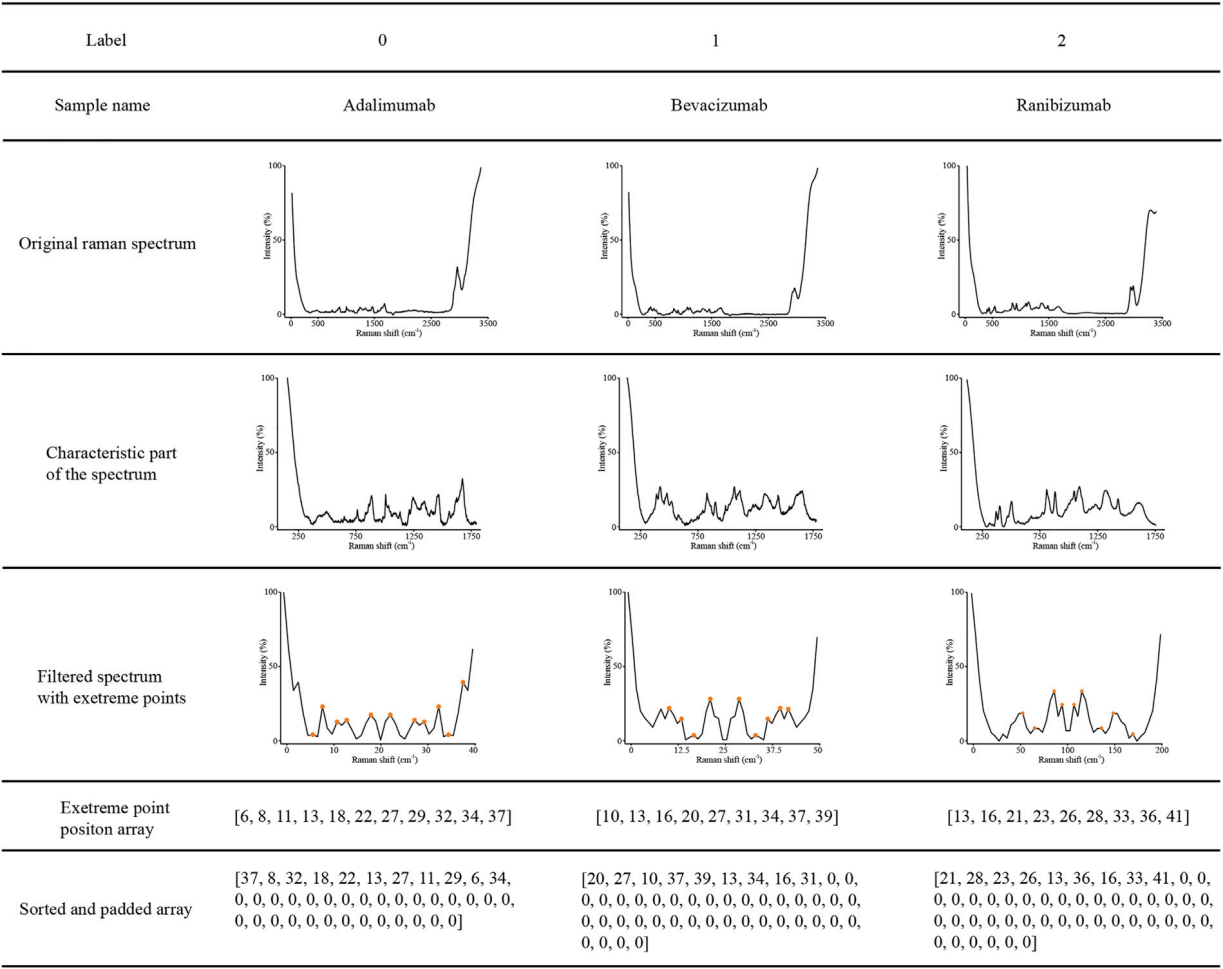
Visualization examples of the original Raman spectra, characteristic region of Raman spectra, characteristic region converted by extreme point transformation, sorted, and padded array of adalimumab, bevacizumab and ranibizumab.

The data visualizations of the extreme point sort transformation of three therapeutic antibodies are shown in Figure 4, which provides insights into the operating mode of the novel data transformation and the conversion process of spectral features. As shown in Figure 4, the original Raman spectrum had a full scan range of 57–3,417 cm^−1^. After interception, a characteristic region with a range of 100–1800 cm^−1^ was drawn as a new subspectrum (Figure 4, Line 4). The target dimensional value for each therapeutic mAb is listed in Table 1, which was used for spectral dimensionality reduction. The filtered spectrum and the extreme points demonstrated that approximately 10 extreme points in each filtered spectrum could be extracted as a sequence array. The elements of the extreme point positions were sorted by their corresponding intensity. Finally, the sorted array was padded with zero to the digitals of the target dimensional value for input of the LSTM network (Figure 4, Line 7).

**TABLE 1:** The primary parameters and training results of the optimized model for the identification of therapeutic monoclonal antibodies.

Label	Dimensional value	Epoch	Loss	Accuracy	Validation loss	Validation accuracy	Threshold
0	40	3	0.0297	0.9975	0.0104	0.9975	0.99
1	47	5	0.0239	0.9929	0.0168	0.9948	0.99
2	49	1	0.3597	0.8839	0.0364	0.9869	0.99
3	42	5	0.0396	0.9875	0.0185	0.9915	0.99
4	48	3	0.0316	0.9909	0.0063	0.9983	0.99
5	46	1	0.4435	0.8605	0.0805	0.9694	0.98
6	41	2	0.0726	0.9774	0.0286	0.9906	0.99
7	35	1	0.4377	0.8644	0.0585	0.9796	0.99
8	42	3	0.0556	0.9821	0.0291	0.9888	0.97
9	53	3	0.0451	0.9853	0.00204	0.9908	0.99
10	39	2	0.0902	0.9729	0.0379	0.9856	0.99
11	47	3	0.0394	0.988	0.0096	0.9973	0.99
12	47	1	0.3216	0.9027	0.0371	0.9877	0.97
13	40	1	0.3225	0.9041	0.0203	0.9903	0.99
14	35	1	0.4184	0.8676	0.0658	0.9794	0.99

### Model Training

A total of 15 models were trained for the extraction of spectral characteristics. The primary parameters and training results of the optimized model for the identification of therapeutic mAbs are listed in Table 1. The optimized target dimensional values were set from 35 to 53. The epoch for training had a range of one to five. The accuracy and validation accuracy of the 15 models ranged from 0.8644 to 0.9975 and 0.9796 and 0.9983, respectively. The optimized threshold values ranged from 0.97 to 0.99.

### Robustness Test

**FIGURE 5:**
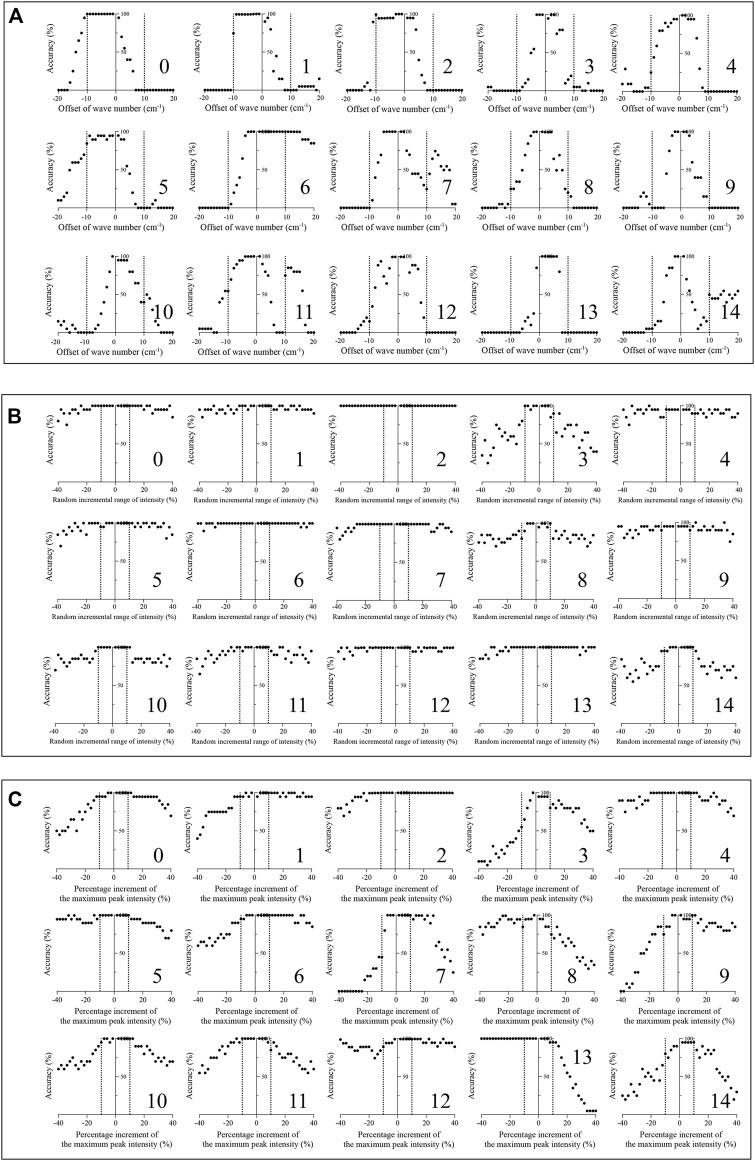
Robustness test of the present algorithm on 15 therapeutic mAbs. **(A)** The classification effect of the present algorithm against simulated spectral peak drift (*n* = 20); **(B)** The classification effect of the present algorithm against simulated spectral noise (*n* = 20); **(C)** The classification effect of the presented method against simulated spectral florescence interference (*n* = 20).

To test the robustness of the algorithm, a total of 36,000 simulated spectra were created manually. The average accuracy results (*n* = 20) are shown in Figure 5A. Fourteen of fifteen therapeutic mAb simulated spectra with an offset of wavenumbers under ±5 cm^−1^ could be identified using the present method to achieve an accuracy over 95%. The simulated spectra of therapeutic mAbs labelled 0, 1, 2, and 6 were correctly identified, while the offsets of the wavenumber were under ±10 cm^−1^. As shown in Figure 5B, all simulated spectra with a random incremental range of 10% or −10% intensity could be identified and achieved an accuracy over 95%. The simulated spectra of therapeutic mAbs labelled two could be correctly identified, while the random incremental range of intensity was in the range of ±40%. As shown in Figure 5C, all simulated spectra with a 10% or −10% increment of the maximum peak intensity could be identified and achieved an accuracy over 95%. The results indicated that the present algorithm had good robustness against spectral peak drift, random noise and fluorescence interference.

### Model Evaluation

**FIGURE 6:**
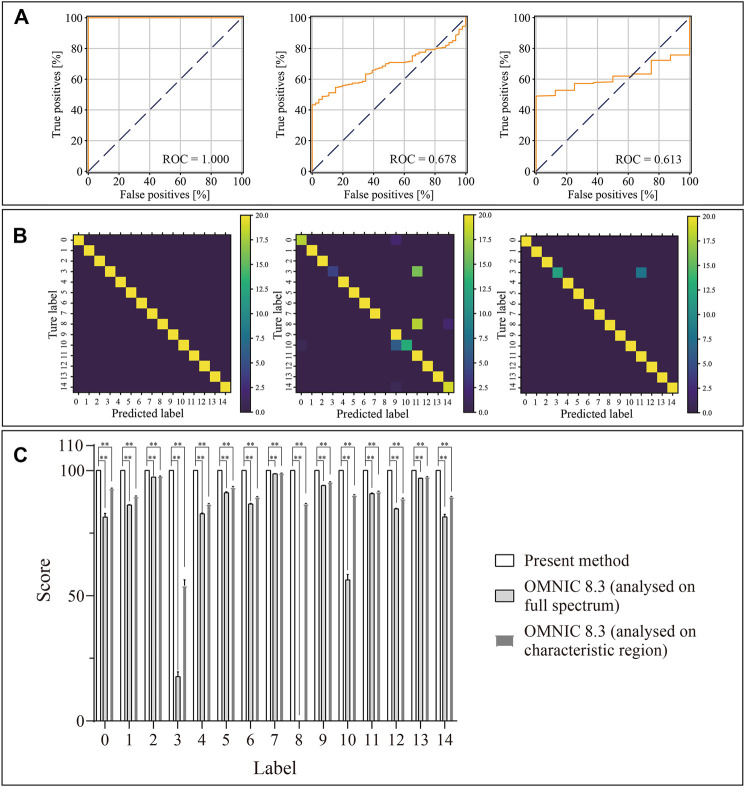
Predicted results of the 15 therapeutic mAbs using the presented method, OMNIC 8.3 software (analysed on the full spectrum) and OMNIC 8.3 software (analysed on the characteristic region). **(A)** Receiver operating characteristic curves of the present algorithm, OMNIC 8.3 software build-in algorithm (analysed on full spectrum) and OMNIC 8.3 software build-in algorithm (analysed on characteristic region); **(B)** Confusion matrixes of the presented method, OMNIC 8.3 software build-in algorithm (analysed on full spectrum) and OMNIC 8.3 software build-in algorithm (analysed on characteristic region) are plotted based on the matching extent between the predicted labels and true labels; **(C)** The matching scores are calculated by model prediction using test samples (*n* = 20). **p < 0.01.

Figure 6 shows the ROC curves of the prediction results, confusion matrixes and similarity scores. As shown in Figure 6A, the areas under the curve (AUC) values of the present algorithm, OMNIC 8.3 software built-in algorithm analysed on the full spectrum and analysed on the characteristic region were 1.000, 0.678 and 0.613, respectively. In the confusion matrix, the diagonal shows the percent of correct prediction records for each therapeutic mAb, and the off-diagonals show the percent of misclassifications for each therapeutic mAb (Figure 6B). The similarity scores of classification for 15 therapeutic mAbs using the present algorithm were 100. The similarity scores for 15 therapeutic mAbs using OMNIC 8.3 (analysed on the full spectrum) were 81.40 ± 1.40, 86.13 ± 0.17, 97.27 ± 0.04, 17.85 ± 1.84, 82.70 ± 0.22, 91.07 ± 0.32, 86.59 ± 0.21, 98.59 ± 0.02, 0, 93.93 ± 0.08, 56.36 ± 2.13, 90.74 ± 0.17, 84.65 ± 0.19, 96.83 ± 0.04 and 81.41 ± 0.98. The similarity scores for 15 therapeutic mAbs using OMNIC 8.3 (analysed in the characteristic region) were 92.76 ± 0.11, 89.58 ± 0.13, 97.68 ± 0.04, 54.07 ± 2.27, 86.49 ± 0.18, 93.20 ± 0.26, 89.33 ± 0.14, 98.83 ± 0.02, 86.63 ± 0.17, 95.32 ± 0.06, 90.10 ± 0.15, 91.47 ± 0.16, 88.71 ± 0.14, 97.36 ± 0.03 and 89.37 + 0.16. All groups compared with the relative present algorithm group had extremely remarkable differences (*p* < 0.001). The results suggested a high classification performance for the present algorithm. These results suggested that the extreme point sort transformation combined with the LSTM network algorithm has an excellent classification performance for Raman-based therapeutic mAb identification.

### Sample Identification

A total of 30 samples, including 15 therapeutic mAb injections and 15 other injections, were tested using the present method. As shown in Table 2, 15 therapeutic mAbs were correctly identified, and 15 non-antibody samples were identified as “not matched”.

**TABLE 2:** Application of the presented method to identify 30 injections, including 15 monoclonal antibodies and 15 other injections.

Sample ID	Category	Sample	Prediction	Similarity score	Matched
1	Therapeutic antibody	Adalimumab	Adalimumab	0.999	Yes
2		Bevacizumab	Bevacizumab	0.998	Yes
3		Ranibizumab	Ranibizumab	0.998	Yes
4		Tocilizumab	Tocilizumab	0.999	Yes
5		Iloeuvumab	Iloeuvumab	0.997	Yes
6		Scuciumab	Scuciumab	0.990	Yes
7		Rituximab	Rituximab	0.992	Yes
8		Trastuzumab	Trastuzumab	0.999	Yes
9		Pertuzumab	Pertuzumab	0.988	Yes
10		Denosumab	Denosumab	0.996	Yes
11		Ixekizumab	Ixekizumab	0.999	Yes
12		Ustekinumab	Ustekinumab	0.997	Yes
13		Guselkumab	Guselkumab	0.986	Yes
14		Almezumab	Almezumab	0.998	Yes
15		Etanercept	Etanercept	0.999	Yes
16	Biochemical drug and biological product	Human immunoglobulin for intravenous injection	NM	—	—
17		Tetanus immunoglobulin injection	NM	—	—
18		Atosiban acetate injection	NM	—	—
19	Chemical drug	Potassium and magnesium aspartate injection	NM	—	—
20		Compound aminobarbital injection	NM	—	—
21		Dopamine hydrochloride injection	NM	—	—
22		Lidocaine hydrochloride injection	NM	—	—
23		Tramadol hydrochloride injection	NM	—	—
24		Aminotoluene acid injection	NM	—	—
25		Epinephrine hydrochloride injection	NM	—	—
26		Bupivacaine hydrochloride injection	NM	—	—
27		Potassium chloride injection	NM	—	—
28		Sodium chloride injection	NM	—	—
29		Invert sugar injection	NM	—	—
30		Sterile water for injection	NM	—	—

NM: not matched.

## Discussion

Raman spectroscopy has been increasingly studied for protein sample analysis, which could indeed offer the possibility of detecting protein bands and chemicals with few preparations and little cost for a few seconds to a few minutes of measurement (Li et al., 2012; Neugebauer et al., 2015; Degardin et al., 2017; Zhang et al., 2019). The injection of therapeutic mAb usually contains antibody proteins and pharmaceutical excipients (Singh et al., 2018; Grilo and Mantalaris, 2019). They are both important components of the injection. The characteristic responses of complex components could be obtained as a spectrum using Raman spectroscopy (Wang et al., 2018). The antibody proteins and pharmaceutical excipients equally contribute to the spectrum. Therefore, it is possible to screen therapeutic mAbs utilizing Raman spectroscopy. However, most Raman peaks of therapeutic mAbs have lower intensities than those of chemical drugs. As a result, the therapeutic mAb spectra could not be identified using the previous algorithm, which is suitable for chemical drugs.

For the analysis method, LSTM networks achieve a better classification effect than other methods (Fan et al., 2019). A bidirectional long short-term memory (Bi-LSTM) network is the process of making any neural network have sequence information in both directions backwards (future to past) or forwards (past to future) (Neishabouri et al., 2021). Bidirectional input flows in two directions, making a Bi-LSTM network preserve the future and past information. In a previous study, 1,200 data points of the Raman spectrum were directly input into the LSTM network, which caused 800 epochs of training (Yu et al., 2021). Excessive training epochs will lead to overfitting of the model. Like sample pretreatment in the field of chemical analysis, preprocessing of spectral data plays an important role in data analysis. In the present study, we provide an extreme point sort transformation to convert the characteristic region of the Raman spectrum to a sequence array. The spectral peak positions and relative intensities, two critical spectral characteristics, were involved in the array. This is an efficient transformation that even the Raman analysis under the influence, and only a few effects would be observed at the identification stage.

FCLs and the softmax function are commonly used in classification models. FCLs are added at the end of the sequence model to make the model end-to-end trainable. The FCLs learn a function between the high-level features given as an output from the previous layers. The softmax function transforms any input values into values between 0 and 1 so that they can be interpreted as probabilities. However, inappropriate parameters of FLCs may lead to overfitting of the model, which will enable counterfeit drugs to be identified as genuine. The result is not accepted in the application of counterfeit drug identification. Therefore, the FLC was only employed in the training process for the fitting model. Then, the model extracted the characteristics from sample spectra followed by calculating the similarity score with standard spectra to classify samples with the threshold value. This step reduces the false-positive rate to avoid identifying the counterfeit drug as genuine.

In summary, we propose a novel extreme port sort transformation combined with an LSTM network algorithm to identify 15 therapeutic mAbs with Raman spectra. The extreme port sort transformation converted the characteristic region of the Raman spectrum into a sequence array containing the original spectral peaks and intensity profile. The characteristic array was extracted from the sequence array by the LSTM network model. The final identification was based on the comparison of the characteristic array. The algorithm was successfully applied in therapeutic mAb identification using an independent test dataset. The algorithm was compared with ThermoFisher OMNIC 8.3 build-in spectrum matching algorithms, and the results indicated that the present algorithm provides a better classification performance.

## Data Availability

The datasets generated for this study can be found here: https://github.com/ttelva2/EPSTLSTM.git.
