# Supplementary material for: Extreme Point Sort Transformation Combined With a Long Short-Term Memory Network Algorithm for the Raman-Based Identification of Therapeutic Monoclonal Antibodies
Source: Front Chem. 2022 Apr 13;10:887960. doi: 10.3389/fchem.2022.887960 (PMC9043956; doi:10.3389/fchem.2022.887960)
Supplement: Supplementary file 1 [file DataSheet1.DOCX]

**Table S1** Information on the 15 monoclonal antibodies involved in this study

| Label | Name | Effect target | Protein format | Manufacturer | Specifications |
| --- | --- | --- | --- | --- | --- |
| 0 | Adalimumab | TNF | Human IgG1 | Abbvie | 40 mg/0.8 mL |
| 1 | Bevacizumab | VEGF | Humanized IgG1 | Roche | 100μg/4 mL |
| 2 | Ranibizumab | VEGF | Humanized IgG1 Fab | Novartis | 10 mg/mL |
| 3 | Tocilizumab | Il-6R | Humanized IgG1 | Roche | 80 mg/4 mL |
| 4 | Evolocumab | PCSK9 | Human IgG2 | Amgen | 140 mg/mL |
| 5 | Secukinumab | IL-17a | Human IgG1 | Novartis | 150 mg/mL |
| 6 | Rituximab | CD20 | Chimeric IgG1 | Roche | 100 mg/10 mL |
| 7 | Trastuzumab | HER2 | Humanized IgG1 | Roche | 440 mg/20 mL |
| 8 | Pertuzumab | HER2 | Humanized IgG1 | Roche | 420 mg/14 mL |
| 9 | Denosumab | RANK-L | Human IgG2 | Amgen | 120 mg/1.7 mL |
| 10 | Ixekizumab | IL-17a | Humanized IgG4 | Lilly | 80 mg/mL |
| 11 | Ustekinumab | IL-12/23 | Human IgG1 | Xian-Janssen | 130 mg/26 mL |
| 12 | Guselkumab | IL-23 p19 | Human IgG1 | Xian-Janssen | 100 mg/mL |
| 13 | Emicizumab | Factor Ixa, X | Humanized IgG4, bispecific | Roche | 30 mg/mL |
| 14 | Etanercept | TNF | Antibody fusion protein | Pfizer | 25 mg/0.47 mL |

**Table S2** The model structure and training parameters

| Layer (type)/Compile | Value | Function (role) | Output Shape | Parameter number |
| --- | --- | --- | --- | --- |
| Embedding (Embedding) | 64 |  | (None, 49, 64) | 3200 |
| LSTM (Bidirectional) | 64 |  | (None, 128) | 66048 |
| Dense1 (Dense) | 32 | Relu (activation) | (None, 32) | 4128 |
| Dropout (Dropout) | 0.35 |  | (None, 32) | 0 |
| Dense (Dense) | 15 | Softmax (Actication) | (None, 15) | 495 |
| Compile |  | Categorical_crossentropy (Loss)  Adam (Optimizer) |  |  |
